# Magnetic interactions in BiFe_0.5_Mn_0.5_O_3_ films and BiFeO_3_/BiMnO_3_ superlattices

**DOI:** 10.1038/srep09093

**Published:** 2015-03-13

**Authors:** Qingyu Xu, Yan Sheng, M. Khalid, Yanqiang Cao, Yutian Wang, Xiangbiao Qiu, Wen Zhang, Maocheng He, Shuangbao Wang, Shengqiang Zhou, Qi Li, Di Wu, Ya Zhai, Wenqing Liu, Peng Wang, Y. B. Xu, Jun Du

**Affiliations:** 1 Department of Physics, Southeast University, Nanjing 211189, China; 2 Key Laboratory of MEMS of the Ministry of Education, Southeast University, Nanjing 210096, China; 3 Collaborative Innovation Center of Suzhou Nano Science and Technology, Soochow University, Suzhou 215123, China; 4 National Laboratory of Solid State Microstructures, Nanjing University, Nanjing 210093, China; 5 Helmholtz-Zentrum Dresden-Rossendorf, Institute of Ion Beam Physics and Materials Research, Bautzner Landstrasse 400, D-01328 Dresden, Germany; 6 Department of Materials Science and Engineering, Nanjing University, Nanjing 210008, China; 7 Department of Physics and Collaborative Innovation Center of Advanced Microstructures, Nanjing University, Nanjing 210093, China; 8 College of Engineering and Applied Sciences and Collaborative Innovation Center of Advanced Microstructures, Nanjing University, Nanjing 210093, China; 9 York-Nanjing Joint Centre, School of Electronic Science and Engineering, Nanjing University, Nanjing 210093, China

## Abstract

The clear understanding of exchange interactions between magnetic ions in substituted BiFeO_3_ is the prerequisite for the comprehensive studies on magnetic properties. BiFe_0.5_Mn_0.5_O_3_ films and BiFeO_3_/BiMnO_3_ superlattices have been fabricated by pulsed laser deposition on (001) SrTiO_3_ substrates. Using piezoresponse force microscopy (PFM), the ferroelectricity at room temperature has been inferred from the observation of PFM hysteresis loops and electrical writing of ferroelectric domains for both samples. Spin glass behavior has been observed in both samples by temperature dependent magnetization curves and decay of thermo-remnant magnetization with time. The magnetic ordering has been studied by X-ray magnetic circular dichroism measurements, and Fe-O-Mn interaction has been confirmed to be antiferromagnetic (AF). The observed spin glass in BiFe_0.5_Mn_0.5_O_3_ films has been attributed to cluster spin glass due to Mn-rich ferromagnetic (FM) clusters in AF matrix, while spin glass in BiFeO_3_/BiMnO_3_ superlattices is due to competition between AF Fe-O-Fe, AF Fe-O-Mn and FM Mn-O-Mn interactions in the well ordered square lattice with two Fe ions in BiFeO_3_ layer and two Mn ions in BiMnO_3_ layer at interfaces.

BiFeO_3_ (BFO) is the most widely studied multiferroic material, due to its above room temperature antiferromagnetism (*T*_N_ ~ 640 K) and ferroelectricity (*T*_CFE_ ~ 1100 K)^1^. BFO has G-type antiferromagnetic (AF) spin structure with spin canting and superimposed cycloidal modulation^2^, and various magnetic anomalies observed at low temperature^3^,^4^. Ion substitution is a widely adopted strategy, suppressing the leakage current and improving the ferroelectricity^5^,^6^, destroying the cycloidal modulation and enhancing the ferromagnetism^7^,^8^, towards the realization of room temperature control of ferromagnetism with an electric field^9^. With magnetic ion substitution in BFO, more complicated magnetic interactions might be included, leading to the observation of cluster spin glass^10^. The clear understanding of exchange interactions between Fe and substituting ions in BFO is important for the comprehensive studies on magnetic properties, e.g., the rather confusing magnetic properties in Mn doped BiFeO_3_, though Mn has been demonstrated to be an effective substituent for ferroelectricity^11^. Mn ions of concentration up to 50% have been doped into BFO by epitaxial growth on (001) SrTiO_3_ (STO) substrates, structure and magnetic properties of BiFe_0.5_Mn_0.5_O_3_ (BFMO) have been studied^12^,^13^,^14^. Strongly enhanced ferromagnetism has been reported by Choi *et al.*^12^, but only negligible weak ferromagnetism has been reported by Bi *et al.*^14^. Furthermore, the superexchange interaction between Fe^3+^ and Mn^3+^ is rather complicated. Ferromagnetic (FM) interaction has been observed between Fe and Mn at the interface of La_0.7_Sr_0.3_MnO_3_ and BFO due to orbital reconstruction^15^, while AF interaction exists at the interface of La_0.5_Ca_0.5_MnO_3_ and BFO without orbital reconstruction^16^.

Both BFO and BMO have distorted perovskite structure with similar pseudocubic lattice constants (3.96 Ǻ for BFO and 3.95 Ǻ for BMO)^17^,^18^, which is very close to that of STO (3.905 Å)^12^. Therefore, in addition to the previously reported solid solution of BFMO, superlattices with alternative BFO and BMO layers are likely to be epitaxially grown on (001) STO substrates, and the inhomogeneous distribution of Fe and Mn will be suppressed^14^. There is only superexchange interaction between Fe and Mn ions at the interfaces along *c* direction, which is expected to facilitate the study of the Fe-O-Mn interaction. In this paper, BFMO films and BFO/BMO superlattices (simply denoted as BFO/BMO) were grown on (001) STO substrates. Spin glass behavior were observed in both samples. The Fe-O-Mn interaction has been confirmed to be AF by X-ray magnetic circular dichroism (XMCD) measurements. Spin glass in BFMO can be categorized as cluster spin glass, while spin glass in BFO/BMO results from competing AF and FM interactions at interfaces.

## Results

**Figure 1:**
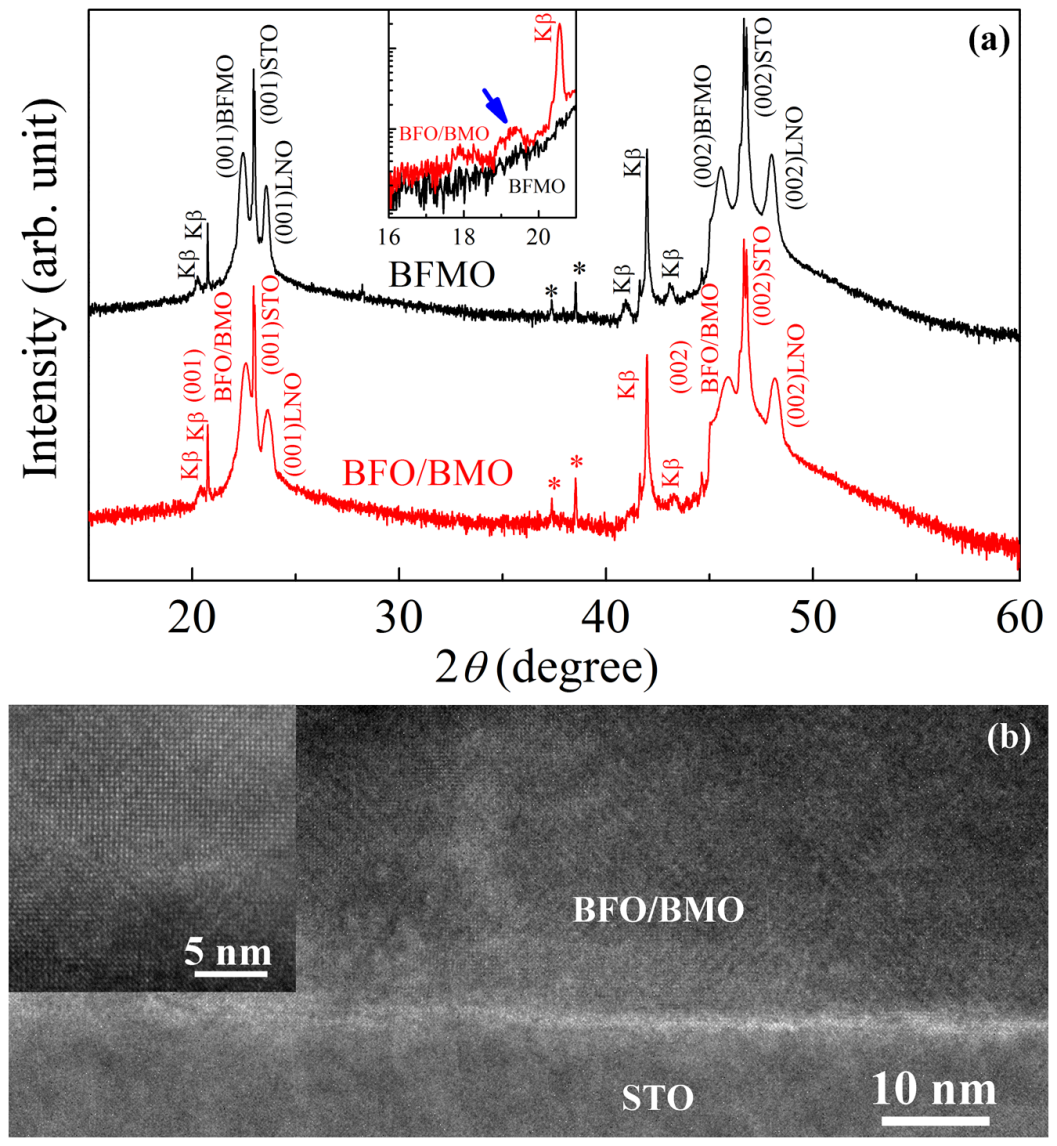
(a) XRD patterns of BFMO and BFO/BMO. Kβ diffraction peaks are indicated. Stars mark the diffraction peaks from STO substrates. Inset shows the diffraction patterns using parallel beam, and the arrow marks the superlattice diffraction peak. (b) Cross-sectional HRTEM image of BFO/BMO, inset is the magnified image.

Figure 1(a) shows the X-ray diffraction (XRD) patterns of BFMO and BFO/BMO with LaNiO_3_ (LNO) as buffer layer in Bragg-Brentano geometry using a D/teX Ultra detector (1D detector). Only (001) and (002) diffraction peaks can be observed, indicating the high (001) orientation, which is due to well matching of lattice constant of BFO (3.96 Ǻ), BMO (3.95 Ǻ) and LNO (3.838 Ǻ) to STO (3.905 Ǻ)^12^,^17^,^18^,^19^. The out-of-plane lattice constants are calculated to be 3.96 Ǻ for BFMO and 3.93 Ǻ for BFO/BMO, respectively. The epitaxial growth of BFO/BMO was further confirmed by a high resolution transmission electron microscope (HRTEM), as shown in Fig. 1(b). However, due to the same crystal structure and similar atomic number of Fe and Mn for the epitaxial layers of BFO and BMO, the interface in the superlattice structure cannot be clearly resolved in the HRTEM images. Considering the pseudo-cubic lattice constant of BFMO of 3.93 Ǻ^13^, the slightly larger *c* lattice constant of BFMO is due to in-plane compression from the STO substrate, and strain relaxation possibly happened in BFO/BMO. The strain due to the lattice mismatch could introduce the contrast variation at the interface between BFO/BMO and STO, shown in Fig. 1(b). According to the phase diagram, BFMO displays predominated orthorhombic structure^20^. The *θ*-2*θ* XRD patterns of BFMO and BFO/BMO were carefully measured in parallel beam geometry using a scintillation detector (the inset of Fig. 1(a)). Due to the close atomic scattering factors of Fe^3+^ and Mn^3+^, the (001) superlattice diffraction peak was hardly resolved. The (002) superlattice peak of BFO/BMO, marked by an arrow, can be clearly seen in the inset, which is absent in the XRD pattern of BFMO. The scintillation detector in parallel beam geometry is much less sensitive than the D/teX Ultra detector in Bragg-Brentano geometry which should reveal the impurity phases. Thus, this superlattice peak cannot be due to impurity phases since it was absent when we used Bragg-Brentano geometry as shown in the main frame of Fig. 1(a). On the other hand, compared with Bragg-Brentano geometry, the parallel beam geometry is more sensitive for the reflection from surfaces and interfaces of films. As shown in the inset of Fig. 1(a), clear observation of the superlattice peak confirms the high concentration of BFO/BMO interfaces. The period of superlattice was calculated using Bragg equation to be about 0.91 nm, which is slightly larger than the designed period (0.79 nm). This is due to the limitation of our PLD system that the layer by layer growth with each layer thickness of 1 unit cell cannot be strictly fulfilled in our work. However, alternative growth of BFO and BMO layers with thickness of roughly 1 pseudo-cubit unit cell provides high concentration of BFO/BMO interfaces and a high interface/bulk ratio, which might facilitate the characterization of Fe-O-Mn superexchange interaction. It has been theoretically predicted the possible checkerboard superstructure in BFO/BMO, which is (110)-oriented superlattice^21^. However, due to the (001) growth direction of our films with alternative (001) BFO and BMO layers, the checkerboard superstructure seems unlikely to be formed.

**Figure 2:**
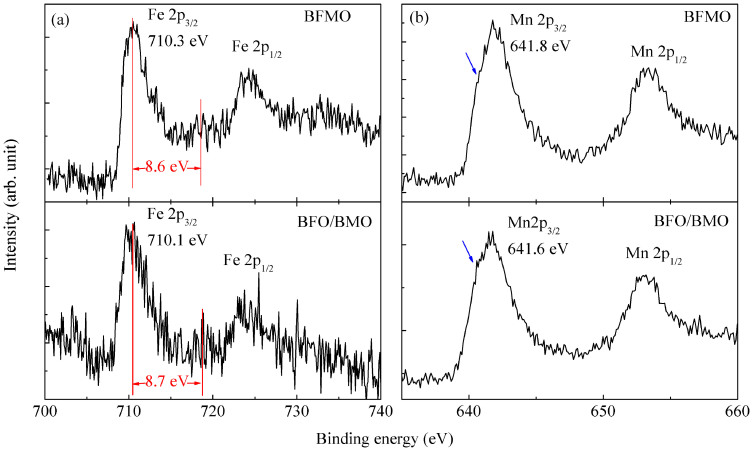
XPS spectra of (a) Fe 2p and (b) Mn 2p for BFMO and BFO/BMO, respectively. Arrows mark the shoulder corresponding to Mn^2+^.

Figure 2 shows the X-ray photoelectron spectroscopy (XPS) core level spectra of Fe 2p and Mn 2p in BFMO and BFO/BMO, calibrated by the C 1s line (284.8 eV) binding energy^22^. The binding energy of Fe 2p_3/2_ is at 710.3 eV for BFMO and 710.1 eV for BFO/BMO. The valence states of Fe in both samples are almost the same after considering the XPS accuracy of ±0.1 eV. However, the decomposition of Fe 2p_3/2_ spectrum into a superposition of symmetric components is questionable, thus it is complicated to obtain the exact concentration of Fe^2+^ and Fe^3+^ 23. A satellite peak can be observed at 8.7 eV for BFO/BMO and 8.6 eV for BFMO above the corresponding principal peak. Due to the different d orbital electron configuration, Fe^2+^ and Fe^3+^ show satellite peak at 6 eV or 8 eV above their 2p_3/2_ principle peaks, respectively^14^. The Fe 2p core level spectra of BFMO and BFO/BMO are similar to those previously reported BFO, confirming that Fe is mainly in +3 valence state^14^. The binding energy of Mn 2p_3/2_ is at 641.8 eV for BFMO and 641.6 eV for BFO/BMO, respectively. A shoulder peak marked by arrow below this energy can be observed in both samples, which originates from a small concentration of Mn^2+^ 14.

**Figure 3:**
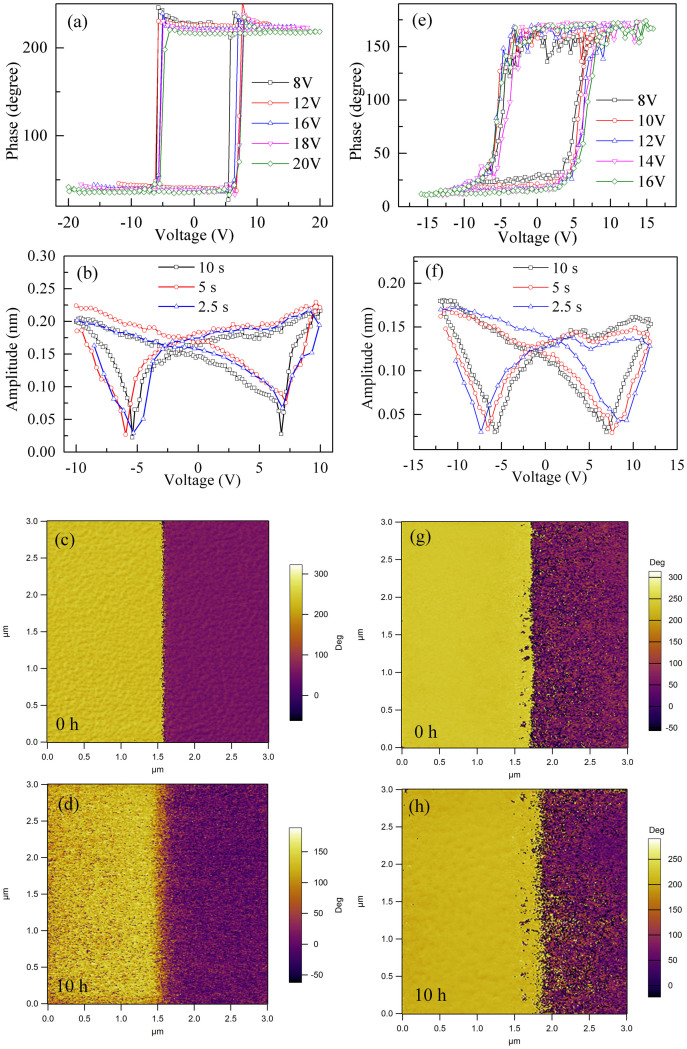
(a) PFM phase and (b) amplitude hysteresis loops for BFMO, (e) PFM phase and (f) amplitude hysteresis loops for BFO/BMO. The domain patterns written by PFM tip in the beginning and after 10 hours for (c) and (d) BFMO, (g) and (h) BFO/BMO.

BFO is a well known multiferroic material with ferroelectricity above room temperature. However, leakage current is a big obstacle for observation of ferroelectric hysteresis loops for both samples. The ferroelectric nature of BFMO and BFO/BMO was characterized at room temperature using piezoresponse force microscopy (PFM), as shown in Fig. 3. The clear local PFM hysteresis loops for both samples suggest their ferroelectricity. It was recently pointed out that similar PFM hysteresis loops were observed in soda-lime glass due to dipoles induced by ionic motion under external electric field^24^. We measured the phase hysteresis loops with various maximum voltages, as shown in Fig. 3(a) and (e), and coercivity of both samples shows little variation. Furthermore, we measured the amplitude hysteresis loops with different frequencies, as shown in Fig. 3(b) and (f). The observed amplitude hysteresis loops for both samples are insensitive to the time periods. Thus, the ferroelectricity in both BFMO and BFO/BMO can be inferred from the PFM results^24^. We further studied the retention of domains written by the PFM tip, as shown in Fig. 3(c), (d), (g) and (h). After 10 hours, negligible changes were observed in domain patterns for both samples.

**Figure 4:**
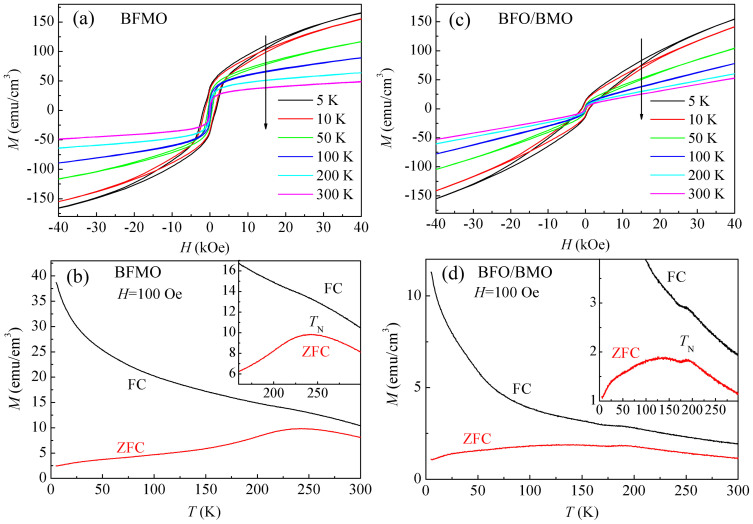
*M*-*H* curves at different temperature for (a) BFMO and (c) BFO/BMO. ZFC and FC *M*-*T* curves for (b) BFMO and (d) BFO/BMO. Insets show the enlarged view.

Figure 4(a) shows the field dependent magnetization (*M*-*H*) curves of BFMO measured at different temperatures. As can be seen, a clear soft FM hysteresis loop can be observed at 300 K, indicating room temperature ferromagnetism. Similar phenomenon has been reported by Choi *et al.*, which was explained by larger strain in ultrathin film^12^. However, others reported only negligible weak ferromagnetism^14^. It should be noted that magnetic properties of pure (001) STO substrate have been checked, the observed weak ferromagnetism is much smaller than the magnetization values of both samples, and can be neglected. The observed magnetization is smaller than that reported by Choi *et al.*^12^, but much larger than that by Bi *et al.*^14^. With decreasing temperature, not only the magnetization increases, but also the coercivity increases drastically, especially below 200 K^25^. The *M*-*H* curves measured at different temperatures for BFO/BMO show similar behavior. The *M*-*H* curves are superposition of paramagnetism and weak ferromagnetism. With applying a maximum magnetic field of 40 kOe, the total magnetization of BFO/BMO is almost the same as that of BFMO, but the FM magnetization of BFO/BMO is much smaller than that of BFMO.

Zero field cooled (ZFC) and field cooled (FC) temperature dependent magnetization (*M*-*T*) curves were measured under 100 Oe from 5 K to 300 K with cooling field of 10 kOe for FC curves, as shown in Fig. 4(b) (BFMO) and (d) (BFO/BMO). A broad peak at around 243 K can be observed in the ZFC *M*-*T* curve for BFMO, but only a kink has been observed in the FC *M*-*T* curve (inset of Fig. 4(b)). With increasing doping concentration of Mn, *T*_N_ of BFO continuously decreases^26^. *T*_N_ of BFMO ceramics decreases to 440 K^27^. Due to positive formation enthalpy of ordered structure of Mn and Fe, the distribution of Mn and Fe is inhomogeneous^14^. As a result, Fe-rich and Mn-rich clusters will form. Three exchange interactions exist in the system, namely Fe-O-Fe, Mn-O-Mn and Fe-O-Mn with different ordering temperature. *T*_N_ of 440 K has been attributed to Fe-O-Fe ordering, which is above the measuring limit of our system. *T*_N_ of BFMO prepared in high pressure with ordered Mn and Fe structure is 270 K^25^, and Du reported the second ordering temperature at 260 K^27^. Thus, we attribute the observed peak at 243 K in *M*-*T* curves to the onset of Fe-O-Mn interaction.

Figure 4(d) shows the ZFC and FC *M*-*T* curves for BFO/BMO. Weaker but sharper peak can be observed at around 190 K in both ZFC and FC *M*-*T* curves, suggesting an AF transition. *T*_N_ of BFO/BMO is smaller than that of BFMO, which might be due to the ordered stacking sequence of Fe and Mn along the *c* direction for BFO/BMO but randomly distribution of Fe and Mn in BFMO. In BFMO, the randomly distributed Fe and Mn have various distances between the neighboring Fe and Mn ions, which might influence the strength of exchange interaction between neighboring Fe and Mn ions, leading to much broader peak in the ZFC *M*-*T* curve of BFMO. For BFO/BMO, Fe and Mn are mostly ordered in out-of-plane direction, leading to a much narrower peak in the ZFC *M*-*T* curve.

**Figure 5:**
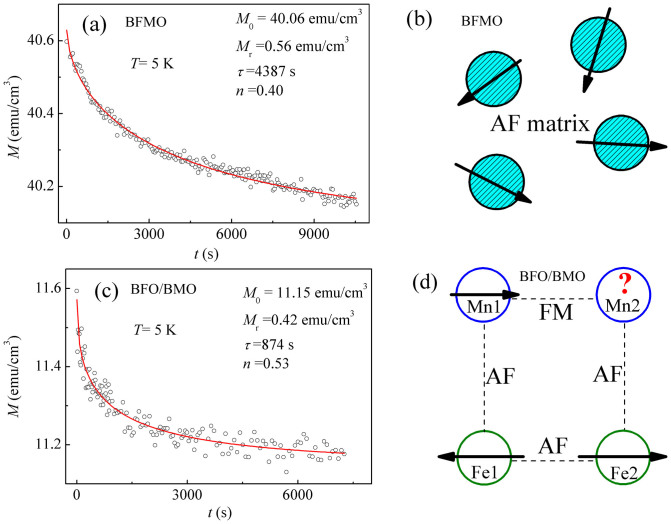
The measured (open symbol) and fitted (solid curve) time dependent remnant magnetization at 5 K for (a) BFMO and (c) BFO/BMO. The schematic structure of (b) cluster spin glass in BFMO, and (d) square lattice of Fe and Mn at interfaces of BFO/BMO. Blue circles in (b) denote Mn-rich clusters in BFMO and arrows denote the net spin directions. Circles in (d) denote Fe and Mn ions with arrows indicating the spin directions.

It is interesting to note that the deviation of FC magnetization from ZFC one at the freezing point (temperature at which ZFC peak occurs) for both samples has been observed. This is a feature, but not exclusive, for the spin glass system^28^,^29^. In both cases of superparamagnet and spin glass, finite dipolar interaction between the spins results in the deviation of FC-ZFC curves at temperature lower than blocking or freezing temperatures and FC magnetization increases continuously as the temperature is lowered^28^. One of the important characteristic features of spin glass is the phenomenon of aging. To confirm the spin glass behavior in BFMO and BFO/BMO, thermo-remnant magnetization (TRM) depending on time was measured at various temperatures below 350 K by cooling the sample in a field of 10 kOe from 350 K to the final temperature, abruptly decreasing field to 500 Oe to measure the time-dependent magnetization. For the magnetic relaxation in spin glass, a stretched exponential decay is expected^28^,^30^, 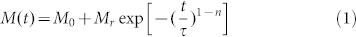
where the glassy component *M*_r_ mainly contribute to the observed relaxation effects. The time constant *τ* and exponent *n* are related to the relaxation rate of spin glass. For 0<*n*<1, it stands for spin glass system^28^,^31^. In equation (1), *M*_0_ is added to account for the nonrelaxed magnetization responding to the applied field of 500 Oe^30^. Figure 5(a) and (c) show typical relaxation curves measured at 5 K for BFMO and BFO/BMO, respectively. Solid curves are the best fitting with equation (1), and fitting parameters are shown. It can be clearly seen that the fitting to experimental data is very well. The parameter of *n* is 0.40 for BFMO and 0.53 for BFO/BMO, which is close to the other spin glass systems^28^,^31^,^32^.

**Figure 6:**
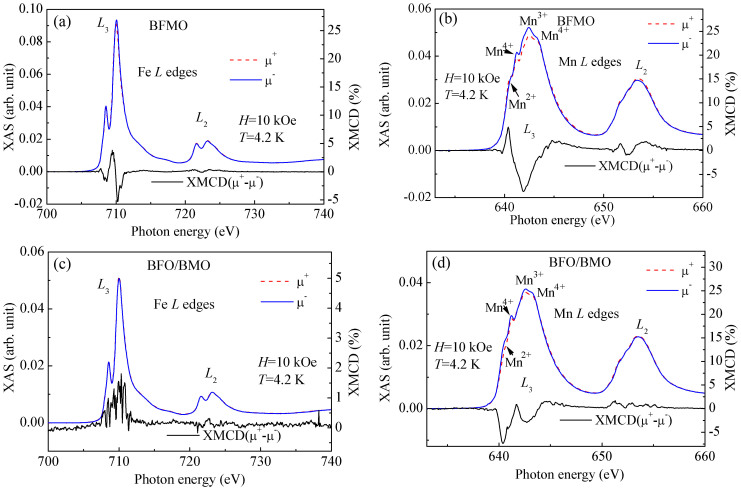
XAS and XMCD spectra of (a) BFMO, Fe *L* edges, (b) BFMO, Mn *L* edges, (c) BFO/BMO, Fe *L* edges, and (d) BFO/BMO, Mn *L* edges, measured at 4.2 K under magnetic field of 10 kOe. Peak positions for Mn^2+^, Mn^3+^ and Mn^4+^ are marked.

Spin glass is generally due to site disorder and lattice frustration, leading to frustrated interactions^33^. Furthermore, if FM clusters are considered as macroscopic spins with competing interactions, spin glass behavior can be observed, termed to cluster spin glass^34^. These spin systems qualitatively exhibit similar and characteristic variations of magnetizations^35^. Thus, XMCD measurements were performed to further clarify the magnetic ordering at low temperatures. Figure 6 shows X-ray absorption spectroscopy (XAS) and XMCD spectra recording at Fe and Mn *L*_2,3_ edges for BFMO and BFO/BMO measured at 4.2 K under a magnetic field of 10 kOe. The line shape of Fe XAS spectra for both BFMO and BFO/BMO are similar to that observed in BFO, confirming the +3 valence state of Fe in both BFMO and BFO/BMO^36^. Furthermore, the line shape is quite different from that of γ-Fe_2_O_3_ or Fe_3_O_4_ which have both *O_h_* and *T_d_* site occupation. Instead, it is close to that of α-Fe_2_O_3_ and GdFeO_3_ with only *O_h_* site occupation, suggesting that Fe ions in both BFMO and BFO/BMO mainly locate at *O_h_* sites^36^,^37^. On the other hand, Mn XAS spectra for both samples possess similar line shape to each other. *L*_3_ peak of Mn locates at around 642.4 eV, suggesting the main valence state of Mn^3+^ 38. Two shoulders can be observed, one at 640.5 eV which can be assigned to Mn^2+^, and the other at 643.3 eV assigned to Mn^4+^ 38. These results suggest the coexistence of Mn^2+^, Mn^3+^ and Mn^4+^ in both samples, which might be due to the inhomogeneous distribution of O^2−^ ions.

In contrast to the similar line shape of Fe and Mn XAS spectra in BFMO and BFO/BMO, XMCD spectra are obviously different. As can be seen in Fig. 6, Fe XMCD spectrum for BFMO is very close to that of γ-Fe_2_O_3_, i.e., having two opposite peaks at *O_h_* and *T_d_* sites, respectively^36^. However, this does not mean that there is large amount of γ-Fe_2_O_3_ or Fe_3_O_4_ impurities, because: i) both BFO and BMO have distorted perovskite structure, and therefore the location of Fe in *T_d_* site seems unreasonable; ii) Fe XAS spectrum in BFMO is quite different from that of γ-Fe_2_O_3_ and Fe_3_O_4_; and iii) XRD results have not shown any peaks of γ-Fe_2_O_3_ and Fe_3_O_4_. The positive formation enthalpy of ordered structure of Mn and Fe leads to inhomogeneous distribution of Mn and Fe^14^. The local variation of Mn doping on structure distortion of FeO_6_ octrahedra with antiparallel alignment of spins leads to opposite signs of XMCD signal at different photon energy. Thus we try to correlate Fe XMCD spectrum to site disorder of Fe ions, since similar XMCD spectrum of Fe *L* edge has been observed in BFO film with high density of domain walls^39^. As shown in Fig. 6(b), a strong peak can be observed in Mn XMCD spectrum, which is much similar to that in BMO film grown on (001) STO substrate^40^, suggesting the formation of Mn-rich clusters with FM Mn-O-Mn interactions. In comparison with weak Fe XMCD signal, the relatively strong Mn signal implies that the enhanced ferromagnetism in BFMO at low temperature is mainly from Mn, instead of Fe.

In contrast to BFMO, BFO/BMO exhibits a small peak in Fe XMCD spectrum, suggesting a weak magnetic contribution from Fe in BFO layers. Previous reports have shown that 4% XMCD signal in BFO/La_0.7_Sr_0.3_MnO_3_ bilayers, which corresponds to a magnetic moment of about 0.6 μ_B_/Fe^15^, and 1% XMCD signal in BFO/La_0.5_Ca_0.5_MnO_3_ corresponding to that of around 0.1 μ_B_/Fe^16^. Accordingly, the observed 1.8% XMCD signal in BFO/BMO can be roughly estimated as 0.2 μ_B_/Fe, which is much larger than the canted moment (0.03 μ_B_/Fe) in bulk BFO^15^. This suggests that the observed 1.8% XMCD does not originate from spin canting in BFO due to the Dzyaloshinskii-Moriya (DM) interaction, while is more likely attributed to the induced spin canting by exchange coupling between Fe and Mn at interfaces^15^,^16^. The weakening of antiferromagnetism and induced weak ferromagnetism in BFO layer at interface have also been observed in BFO/CoFe and BFO/CoFeB due to exchange coupling^41^,^42^. For Mn XMCD spectrum in the BFO/BMO, we have found a splitting at *L*_3_ edge, different from the single peak observed in BMO^40^. This discrepancy could be explained as that BMO layer is not strictly one unit cell thick, thus Mn ions have different neighboring environments. For instance, Mn ions inside BMO layer have only the nearest neighboring Mn ions, while Mn ions at interfaces have the nearest neighboring Fe ions, leading to various distortion on MnO_6_ octahedral. These two different locations of Mn ions possibly lead to the splitting at *L*_3_ edge. The same sign of split XMCD peaks suggests that Mn spins tend to align parallel, confirming the FM interaction of Mn-O-Mn. Comparing Fe and Mn XMCD spectra in BFO/BMO, their opposite sign suggests an antiparallel alignment of the corresponding magnetic moments, confirming the AF exchange interaction of Fe-O-Mn at interfaces. The Mn spins in BMO layer tend to align parallel to each other due to FM interaction between Mn ions. Thus the spins of neighboring Fe ions in BFO layer will be forced to align parallel through the AF interaction of Fe-O-Mn at interfaces. Together with AF interaction between neighboring Fe ions in BFO layer, spin canting might be enhanced, leading to enhanced weak ferromagnetism.

## Discussion

The XMCD results on BFMO clearly demonstrated the formation of FM Mn-rich clusters in AF Fe-rich matrix, indicating the cluster spin glass^34^, as the schematic diagram shown in Fig. 5(b). Considering the layered growth structure of BFO/BMO, a square lattice has been formed at interface with two Fe ions in BFO layer and two Mn ions in BMO layer, as shown in the schematic structure in Fig. 5(d). Due to AF exchange interaction, the spins of two neighboring Fe ions, Fe1 and Fe2 in BFO layer, align antiparallel. The AF exchange interaction between neighboring Fe and Mn at interface will force the spins of Mn ions to align antiparallel to the neighboring Fe ions, thus the spins of neighboring Mn ions will be forced to align antiparallel to each other. However, FM exchange interaction between neighboring Mn ions will force the spins to align parallel to each other, leading to high frustration. Geometrical frustration is generally observed in triangle lattices without disorder that has AF exchange interaction between the nearest neighboring magnetic ions. Magnetic frustration might also be realized in the well ordered square lattice with finely tuned AF and FM interactions^33^. The spin glass in BFO/BMO can be understood by the competing AF (Fe-O-Fe in BFO, Fe-O-Mn at interface) and FM (Mn-O-Mn in BMO) exchange interactions at interfaces with well ordered square lattices similar to geometrical frustration in triangle lattices^33^.

In summary, comparative structural and magnetic studies have been performed on multiferroic BFMO and BFO/BMO prepared by PLD on (001) STO substrates. The ferroelectricity at room temperature for both samples has been inferred from the observation of PFM hysteresis loops and electrical writing of ferroelectric domains. Irreversibility in FC and ZFC *M*-*T* curves has been observed in both samples, with a cusp at around 243 K for BFMO and 190 K for BFO/BMO in ZFC curves. The decay of thermo-remnant magnetization with time confirms the spin glass behavior. XMCD measurements confirm the AF interaction of Fe-O-Mn. Spin glass behavior in BFMO has been classified to cluster spin glass due to Mn-rich FM clusters embedded in AF matrix. Spin glass behavior in BFO/BMO is due to competition among AF Fe-O-Fe interaction in BFO, AF Fe-O-Mn interaction at interface, and FM Mn-O-Mn interaction in BMO in the well ordered square lattices at interfaces of BFO and BMO.

## Methods

BFMO, BFO and BMO ceramic targets were prepared by tartaric acid modified sol-gel method^43^. BFMO and BFO/BMO films were deposited on (001) STO substrates by pulsed laser deposition (PLD) system with a KrF eximer laser of 248 nm and a repetition rate of 5 Hz. Laser energy was 300 mJ and target-to-substrate distance was kept at 5 cm. Substrate temperature *T*_s_ was kept at 750°C with oxygen pressure *P*_O2_ of 2 Pa. BFO/BMO was prepared by alternatively ablating BFO and BMO targets with 5 pulses for each layer and the stacking sequence was repeated 50 times. The thickness of about one pseudo-cubic unit cell was deposited by 5 laser pulses, estimated from the average growth speed of BFO film. For BFMO, 500 pulses were selected. After deposition, both BFMO and BFO-BMO were annealed for 0.5 h at 550°C and cooled down to room temperature in an oxygen pressure of 1 × 10^5^ Pa. The film thickness was determined by the cross-sectional scanning electron microscopy (SEM, FEI) images to be 34 nm for BFO/BMO and 25 nm for BFMO. For magnetization measurements, the films were directly deposited on STO surface, while a LNO buffer layer with thickness of about 30 nm was deposited first at *T*_s_ = 880°C and *P*_O2_ = 40 Pa for the other measurements.

The crystal structure of films was examined by XRD with Cu *K*α radiation (Rigaku Smartlab3). The valence states of Fe and Mn were characterized by XPS (ThermoFisher SCIENTIFIC) with Al Kα X-ray source (h*ν* = 1486.6 eV). The cross-sectional specimen for HRTEM was prepared by mechanical polishing followed by argon ion milling. The thinned sample was examined using a JEM-200CX. The surface morphology and ferroelectric domains were characterized by scanning probe microscopy (SPM, Asylum Research Cypher). Temperature dependent magnetic properties were carefully measured by a commercial SQUID-VSM (Quantum Design) from 5 K to 300 K. XAS measurements were performed at the beam line UE46/PGM-1 at BESSY II (Helmholtz-Zentrum Berlin) with a circular degree of polarization of around 90%. The spectra were acquired and normalized to the incident beam in total electron yield (TEY) mode by recording the sample drain current as a function of photon energy. Right-handed (μ^+^) and left-handed (μ^−^) circularly polarized XAS spectra were obtained by reversing photon helicity under *H* = 10 kOe. The field is parallel to the beam, and the beam is perpendicular to the surface plane of our samples. XMCD spectrum was obtained as (μ^+^−μ^−^) and normalized to the maximum peak intensity of XAS [(μ^+^ + μ^−^)/2].

## Author Contributions

Q.X. and J.D. initiated the study. Y.S. prepared the samples by PLD. M.K., Y.W. and S. Z. performed the XAS and XMCD measurements. S.W. and P.W. performed the HRTEM investigation. Y.C. and D.W. performed the XPS measurements. X.Q. and D.W. performed the SPM measurements. M.H., J.D. and S.Z. performed the magnetic measurements. Q.X., Y.W., W.Z., S.Z., Q.L., Y.Z., W.L. and Y.B.X. analyzed the XAS and XMCD results. Q.X., S.Z., Q.L., W.L., Y.B.X. and J.D. prepared the manuscript. All the authors contributed to discussions of the project and reviewed the manuscript.
